# Angiotensin-converting enzyme insertion/deletion gene polymorphism and the progression of cerebral microbleeds

**DOI:** 10.3389/fneur.2023.1230141

**Published:** 2023-10-12

**Authors:** Cindy W. Yoon, Jonguk Kim, Young Ju Suh, Byeong C. Kim, Young Chul Youn, Jee Hyang Jeong, Hyun Jeong Han, Seong Hye Choi

**Affiliations:** ^1^Department of Neurology, Inha University School of Medicine, Incheon, Republic of Korea; ^2^Department of Biomedical Sciences, Inha University School of Medicine, Incheon, Republic of Korea; ^3^Department of Neurology, Chonnam National University Medical School, Gwangju, Republic of Korea; ^4^Department of Neurology, Chung-Ang University College of Medicine, Seoul, Republic of Korea; ^5^Department of Neurology, Ewha Womans University School of Medicine, Seoul, Republic of Korea; ^6^Department of Neurology, Myongji Hospital, Hanyang University College of Medicine, Goyang, Republic of Korea

**Keywords:** cerebral microbleeds (CMB), cerebral small vessel disease (CSVD), angiotensin-converting enzyme, polymorphism, insertion/deletion polymorphism

## Abstract

**Background and purpose:**

The angiotensin-converting enzyme (ACE) insertion (I)/deletion (D) polymorphism has been studied as a genetic candidate for cerebral small vessel disease (CSVD). However, no previous study has evaluated the relationship between the ACE I/D polymorphism and cerebral microbleed (CMB), an important CSVD marker. We evaluated the association between ACE I/D polymorphisms and 2-year changes in CMBs.

**Methods:**

The CHALLENGE (Comparison Study of Cilostazol and Aspirin on Changes in Volume of Cerebral Small Vessel Disease White Matter Changes) database was analyzed. Of 256 subjects, 186 participants who underwent a 2-year follow-up brain scan and ACE genotyping were included. Our analysis was conducted by dividing the ACE genotype into two groups (DD vs. ID/II) under the assumption of the recessive effects of the D allele. A linear mixed-effect model was used to compare the 2-year changes in the number of CMBs between the DD and combined ID/II genotypes.

**Results:**

Among 186 patients included in this study, 24 (12.9%) had the DD genotype, 91 (48.9%) had the ID genotype, and 71 (38.2%) had the II genotype. Baseline clinical characteristics and cerebral small vessel disease markers were not different between the two groups (DD vs. ID/II) except for the prevalence of hypertension (DD 66.7% vs. ID/II 84.6%; *p* = 0.04). A multivariate linear mixed-effects model showed that the DD carriers had a greater increase in total CMB counts than the ID/II carriers after adjusting for the baseline number of CMBs, age, sex, and hypertension (estimated mean of difference [standard error (SE)] = 1.33 [0.61]; *p =* 0.03). When we performed an analysis of cases divided into deep and lobar CMBs, only lobar CMBs were significantly different between the two groups (estimated mean of difference [SE] = 0.94 [0.42]; *p =* 0.02).

**Conclusion:**

The progression of CMBs over 2 years was greater in the ACE DD carriers compared with the combined II/ID carriers. The results of our study indicate a possible association between the ACE I/D polymorphism and CMB. A study with a larger sample size is needed to confirm this association.

## Introduction

Cerebral small vessel disease (CSVD) is a disorder of the small perforating arterioles, capillaries, and venules of the brain (1). Clinically, CSVD is a major contributor to stroke (both ischemic and hemorrhagic) and dementia. CSVD causes about a quarter of ischemic strokes and most hemorrhagic strokes, is the most common cause of vascular dementia, and often co-occurs with Alzheimer’s disease (1). CSVD is associated with aging and vascular risk factors, especially hypertension (HTN) (2). Neuroimaging markers of CSVD include lacunes, white matter hyperintensities (WMH), cerebral microbleeds (CMBs), and enlarged perivascular spaces (3).

Angiotensin-converting enzyme (ACE) is a key regulator of the renin-angiotensin system that converts inactive angiotensin-I (Ang-I) to active angiotensin-II (Ang-II), which causes vasoconstriction and increases sodium and water retention, leading to increased blood pressure. The gene encoding ACE is located on the long arm of chromosome 17 (17q23) (4). The ACE insertion (I)/deletion (D) polymorphism is known to influence ACE levels, and the D allele is associated with higher ACE level and activity (4). The ACE I/D polymorphism has been studied as a genetic candidate for CSVD because Ang-II has a critical role in HTN, a major risk factor for CSVD, and can affect cerebral circulation by promoting oxidative stress, leading to vascular damage and dysfunction (5).

Although results have not been consistent across studies, an association between the ACE I/D polymorphism and CSVD markers including WMH or lacunar infarct has been suggested (6–9). CMB is a clinically important CSVD marker of a bleeding-prone microangiopathy that is related to the risk of hemorrhagic stroke (10). A recent meta-analysis indicated that the ACE I/D polymorphism is associated with a risk of hemorrhagic stroke (11). However, no previous study has evaluated the relationship between the ACE I/D polymorphism and CMB. This study investigated the impact of the ACE I/D polymorphism on the progression of CMBs.

## Methods

### Study participants

This study was a sub-analysis of the CHALLENGE (Clinicaltrials.gov; Unique identifier: NCT01932203) trial, a multicenter, double-blind, randomized controlled trial that enrolled participants aged 50–85 years with CSVD (12). A diagnosis of CSVD was established based on the presence of at least one lacune and moderate to severe WMH, according to the modified Fazekas criteria for periventricular WMH with a cap or rim of ≥5 mm and deep WMH with a maximum diameter of ≥10 mm (13). The main objective of the trial was to compare the effects of cilostazol and aspirin on changes in the WMH volume over 2 years. Between July 2013 and August 2016, 282 participants were screened for eligibility, of whom 256 were randomly assigned to the cilostazol or aspirin group using a permuted block randomization method (12). Out of 256 CHALLENGE subjects, 186 participants who underwent a 2-year follow-up magnetic resonance imaging (MRI) and ACE I/D genotyping were included. A comparison between the included and excluded subjects is shown in Supplementary Table S1. There were no significant differences between the two groups.

The Institutional Review Boards of the participating centers approved this study. The approval number of the affiliated center of the corresponding author (SC) was 2013–03-006. Written informed consent was obtained from all potential participants prior to enrollment.

### Genotyping of the ACE I/D polymorphism

DNA was extracted from a 2 mL blood sample from each participant. To isolate the buffy coat from the 2 mL blood sample, blood collected in an EDTA-containing tube was centrifuged at 1500 × g for 10 min. The buffy coat was carefully extracted using a fine-tipped pipette and stored at −80°C for further analysis. A QuickGene DNA Whole Blood Kit S (Kurabo, Osaka, Japan) was used to extract DNA from the buffy coat according to the manufacturer’s instructions. The genotyping analysis was performed after the CHALLENGE study was completed.

Genotyping of the ACE I/D polymorphism was performed by DNA direct sequencing. PCR was used to amplify the ACE fragments using UCSC In-Silico PCR.^1^ The final volume of the PCR test sample was 10 μL, consisting of 10 ng of DNA, 0.5 nM of each primer pair, 0.25 mM dNTPs, 3 mM MgCl_2_, 1 μL 1× reaction buffer, and 0.25 U Taq DNA polymerase (Intron Biotechnology, Seongnam-Si, Gyeonggi-do, Korea). The region of intron 16 was amplified using PCR primers (forward; 5′-GAGAGGAGAGAGACTCAAGC-3′, reverse; 5′-AGCCTGGTTGATGAGTTC-3′) designed by DNA LINK Inc. The PCR conditions used were as follows: initial denaturation at 95°C for 10 min, followed by 35 cycles of denaturation at 95°C for 30 s, annealing at 60°C for 1 min, initial extension at 72°C for 1 min, and final extension at 72°C for 10 min. The PCR products were purified using a MultiScreen384-PCR Filter Plate (Millipore, Billerica, MA, United States). The purified products were then sequenced using a BigDye Terminator Cycle Sequencing Kit and an ABI 3730xl automated sequencer (Applied Biosystems, Foster City, CA, United States). The sequencing primers were the same as those used for the PCR amplification. Mutation analyzes were performed using Phred, Phrap, Consed, and Polyphred 5.04 software.^2^

### Imaging markers

Brain MRI data including an axial T2∗-weighted gradient-echo sequence (4-mm slice thickness with no interslice gap) were acquired using a 3.0 Tesla MR scanner. The same scanner and sequence were used for the baseline and follow-up MRI. CMBs were defined as lesions with a diameter ≤ 10 mm and rated using the Microbleed Anatomical Rating Scale (14). Two experienced neurologists blinded to the clinical information counted the number of CMBs on gradient-echo MRI images. Pearson’s correlation coefficient of agreement on the number of CMBs between the two neurologists was 0.958 (95% confidence interval 0.809–0.989; *p* < 0.001). The two neurologists reached a consensus after discussing cases where there was an initial disagreement. CMBs were categorized as deep (basal ganglia, thalamus, internal/external capsule, corpus callosum, deep/periventricular white matter, and brainstem) or lobar (frontal, parietal, temporal, occipital, and insular cortices). The decision on CMB progression was made in a blind manner without access to any other clinical information.

### Statistical analysis

Referring to previous research results (7, 15), our analysis was conducted by dividing the ACE I/D genotype into two groups (DD vs. ID/II) under the assumption of the recessive effects of the D allele. The baseline characteristics were compared between the DD and combined ID/II genotypes using the chi-square test for categorical variables and the Student’s *t*-test or the Mann–Whitney *U*-test for continuous variables. The change in the number of CMBs and the proportion of patients with CMB progression (defined as an increase in the number of CMBs ≥1) during the 2-year follow-up period were compared using the Mann–Whitney *U*-test and the chi-square test. We used a linear mixed-effects model with a random subject effect to estimate and compare changes in the number of CMBs over 2 years. To assess the trend in each group, linear mixed-model analyzes were performed separately using time (baseline and 2-year follow-up visit) as a predictor. To determine the impact of the ACE I/D genotype on the longitudinal changes in CMB counts, we explored the interaction between the ACE I/D genotype and time (ACE I/D genotype × time) adjusted for the baseline number of CMBs, age, sex, and HTN.

## Results

Among 186 included patients, 24 (12.9%) had the DD genotype, 91 (48.9%) had the ID genotype, and 71 (38.2%) had the II genotype. We compared the DD genotype with the combined ID/II genotypes assuming the recessive effect of the D allele. The baseline clinical characteristics were not different between the two groups except for the prevalence of HTN (DD 66.7% vs. ID/II 84.6%; *p* = 0.04) (Table 1). There was no difference in baseline CSVD markers, including baseline number of CMBs, between the two groups. After 2 years of follow-up, the proportion of patients with CMB progression (defined as an increase in the number of CMBs by ≥1) was 54.2% (13/24) for the DD genotype and 48.8% (79/162) for the ID/II genotype (*p* = 0.67). The median (interquartile range) increase in the number of CMBs over 2 years was 1 (0–3) for the DD genotype and 0 (0–1) for the ID/II genotype (*p* = 0.38).

**Table 1:** Comparison of the baseline characteristics according to the angiotensin-converting enzyme (ACE) gene insertion (I)/deletion (D) polymorphism.

	DD (*n* = 24)	ID/II (*n* = 162)	*p-*value
Age, years	75.1 (7.5)	73.1 (6.6)	0.16
Female	15 (62.5)	106 (65.4)	0.82
Hypertension	16 (66.7)	137 (84.6)	0.04
Diabetes	9 (37.5)	63 (38.9)	1.00
Hyperlipidemia	10 (41.7)	81 (50.6)	0.51
Current Smoking	1 (4.2)	11 (6.8)	1.00
Body mass index, kg/m^2^	24.7 (2.6)	24.7 (3.1)	0.99
Antiplatelet medication			0.27
Aspirin	10 (41.7)	90 (55.6)	
Cilostazol	14 (58.3)	72 (44.4)	
Follow-up, years	1.96 (0.22)	1.97 (0.17)	0.58
Baseline CSVD markers			
WMH volume, mL	45.6 (31.6–52.1)	33.7 (23.6–47.6)	0.07
Number of lacunes	6 (2–12)	5 (2–10)	0.53
Number of CMBs			
Total	1 (1–12)	2 (0–7)	0.49
Deep	1 (0–8)	1 (0–4)	0.55
Lobar	1 (0–4)	0 (0–2)	0.36

The values are presented as percentages (%), mean (standard deviation), or median (interquartile range).

CSVD, cerebral small vessel disease; WMH, white matter hyperintensities; CMB, cerebral microbleed.

**Figure 1:**
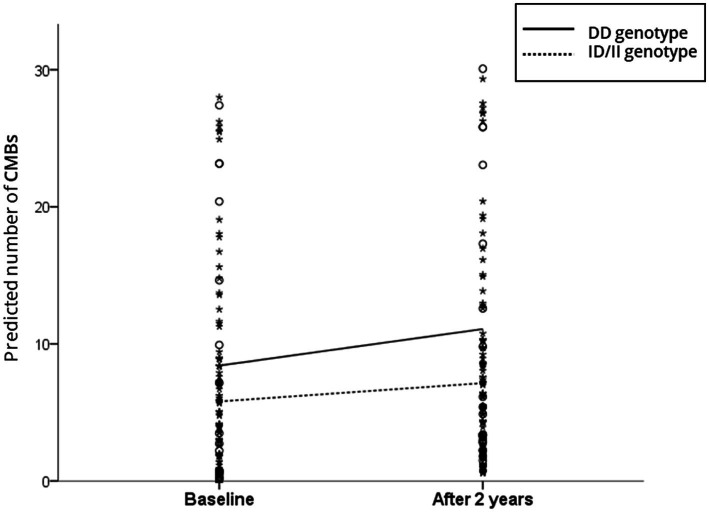
Scatterplot of the predicted number of cerebral microbleeds (CMBs) according to the angiotensin-converting enzyme (ACE) gene insertion (I)/deletion (D) polymorphism. The solid and dotted lines indicate the linear regression model of patients with the DD and ID/DD genotypes, respectively. The analysis controlled for the baseline number of CMBs, age, sex, and hypertension.

In the linear mixed-effect model, which tested the effect of the ACE I/D genotype × time interaction on changes in CMB counts, the DD carriers had a much greater increase in total CMB counts than the ID/II carriers after adjusting for the baseline number of CMBs, age, sex, and HTN (estimated mean of difference [standard error (SE)] = 1.33 [0.61]; *p =* 0.03) (Table 2). Figure 1 shows the estimated effect of the ACE I/D genotype on the longitudinal changes in the number of total CMBs over a 2-year follow-up period. In the analysis using CMBs divided into deep and lobar CMBs, only lobar CMBs showed a significant difference between the two groups (estimated mean of difference [SE] = 0.94 [0.42]; *p =* 0.02) (Table 2).

**Table 2:** Comparison of the longitudinal changes in the number of cerebral microbleeds according to the angiotensin-converting enzyme (ACE) gene insertion (I)/deletion (D) polymorphism.

Changes in the number of CMBs over the 2-year follow-up period^a^				
	DD	ID/II	Differences between DD and ID/II (ID/II as a reference)	
	Estimated mean (SE)	Estimated mean (SE)	Estimated mean (SE)	*P-*value
Total	2.67 (0.93)	1.34 (0.18)	1.33 (0.61)	0.03
Deep	1.04 (0.36)	0.60 (0.10)	0.44 (0.29)	0.14
Lobar	1.63 (0.77)	0.69 (0.11)	0.94 (0.42)	0.02

CMBs, cerebral microbleeds; SE, standard error.

a Results of a linear mixed model adjusted for the baseline number of CMBs, age, sex, and hypertension.

## Discussion

CMB is an important CSVD marker associated with the risk of stroke, cognitive decline, and depression (10, 16–19). In this longitudinal study, we compared the progression of CMBs according to the ACE I/D polymorphism. This is the first study to investigate the relationship between the ACE I/D polymorphism and CMBs. Our major finding was that the progression of CMBs over 2 years was greater in the DD homozygote carriers compared with the combined II/ID carriers.

The ACE I/D polymorphism affects the level and activity of ACE, which converts Ang-I to Ang-II (4). The formation of CMB is caused by the structural weakening and endothelial dysfunction of the microvasculature (20). Ang-II promotes oxidative stress in the cerebral vasculature, which leads to endothelial dysfunction, increased blood–brain barrier permeability, inflammation, and vascular structural damage (5). These effects of Ang-II on the cerebral microvasculature can cause vascular leakage from vulnerable small vessels, which leads to the formation of CMB. Because the ACE level and activity are known to be higher in the DD genotype than in the other two genotypes (4), this could be a possible mechanism for the more pronounced CMB progression in the DD carriers.

HTN is a major risk factor for CMB formation (10) and Ang-II plays a role in increasing the blood pressure. However, in our study, the prevalence of HTN was higher in the II/ID carriers, but the progression of CMBs was more pronounced in the DD carriers. This suggested that the more pronounced CMB progression in DD carriers was not due to the influence of the D allele on HTN incidence. However, even in patients with HTN, the progression of CMBs may differ depending on the actual blood pressure control status and blood pressure variability (21, 22). One of the limitations of our study was that we did not have detailed information on the level of blood pressure control and blood pressure variability.

The DD homozygote carriers had a significantly greater increase in the number of total and lobar CMBs than the ID/II carriers, but no significant difference in the number of deep CMBs. The deep perforating arteries are affected in deep CMBs, whereas the cortical and leptomeningeal arteries are affected in lobar CMBs. Regarding the inconsistent results between the deep and lobar CMBs, the effect of Ang-II on the vasculature might differ depending on the vascular location. However, it should be noted that the prevalence of HTN was significantly higher in the ID/II carriers compared with the DD carriers (84.6% vs. 66.7%) in our study. Although HTN was adjusted for in the analysis, the higher prevalence of HTN in the ID/II carriers might offset the impact of the DD genotype on the progression of CMBs because HTN has a strong influence, especially on the formation of deep MBs.

Our data should be interpreted with caution due to the small sample size. A study with a larger sample size is needed to test and confirm the genetic association. Furthermore, only Koreans were included, which could limit the generalizability of our results. Also, because our patient group consisted of older individuals with relatively severe pre-existing CSVD, this limits the generalization of our results. Finally, although an adjustment for confounding factors was made in the multivariate analyzes, differences in the prevalence of HTN between the genotypes were also an important limitation of our study. Finally, as mentioned earlier, we did not have detailed information on the level of blood pressure control or blood pressure variability.

## Conclusion

In this study, the progression of CMBs over 2 years was greater in the ACE DD carriers compared with the combined II/ID carriers. Our results suggest that the ACE I/D polymorphism is associated with CMBs. Further studies with larger multiethnic samples are needed to confirm this association.

## Data availability statement

The raw data supporting the conclusions of this article will be made available by the authors, without undue reservation.

## Ethics statement

The studies involving humans were approved by Inha University Hospital Institutional Review Board. The studies were conducted in accordance with the local legislation and institutional requirements. Written informed consent for participation in this study was provided by the participants' legal guardians/next of kin.

## Author contributions

CY, JK, and SC contributed to the conception and design of the study. CY, JK, BK, YY, JJ, HH, and SC collected the data and organized the database. CY and YS performed the statistical analysis. CY and JK wrote the first draft of the manuscript. SC reviewed and edited the manuscript. All authors contributed to the article and approved the submitted version.

## Footnotes

^1^ http://genome.ucsc.edu/cgi-bin/hgPcr?command=start

^2^ http://droog.mbt.washington.edu/PolyPhred.html
