# Supplementary material for: Angiotensin-converting enzyme insertion/deletion gene polymorphism and the progression of cerebral microbleeds
Source: Front Neurol. 2023 Oct 12;14:1230141. doi: 10.3389/fneur.2023.1230141 (PMC10602736; doi:10.3389/fneur.2023.1230141)
Supplement: Supplementary file 1 [file Table_1.DOCX]

**Supplementary Table 1. Comparison between the included and excluded patients**

|  | Included patients (*n* = 186) | Excluded patients (*n* = 70) | *P* |
| --- | --- | --- | --- |
| Age, years | 73.3 (6.8) | 75.1 (6.3) | 0.08 |
| Sex, female | 121 (65.1) | 42 (60.0) | 0.47 |
| Hypertension | 153 (82.3) | 56 (80.0) | 0.72 |
| Diabetes | 72 (38.7) | 34 (48.6) | 0.16 |
| Dyslipidemia | 91 (49.5) | 35 (50.7) | 0.89 |
| Current Smoking | 12 (6.5) | 7 (10.0) | 0.42 |
| Body mass index, kg/m^2^ | 24.7 (3.1) | 24.5 (3.5) | 0.55 |
| Baseline number of CMBs |  |  |  |
| Deep | 1 (0-4) | 1 (0-3) | 0.68 |
| Lobar | 1 (0-2) | 0 (0-3) | 0.66 |
| Total | 2 (0-7) | 2 (0-7) | 0.59 |

The values are presented as the number (%), mean (SD), or median (IQR). CMB, cerebral microbleed; SD, standard deviation; IQR, interquartile range.
